# Oral Lichen Planus and Oral Squamous Cell Carcinoma share key oncogenic signatures

**DOI:** 10.1038/s41598-022-24801-6

**Published:** 2022-11-30

**Authors:** Cristóvão Antunes de Lanna, Beatriz Nascimento Monteiro da Silva, Andreia Cristina de Melo, Martín H. Bonamino, Lísia Daltro Borges Alves, Luis Felipe Ribeiro Pinto, Abel Silveira Cardoso, Héliton Spíndola Antunes, Mariana Boroni, Daniel Cohen Goldemberg

**Affiliations:** 1 Laboratory of Bioinformatics and Computational Biology, Division of Experimental and Translational Research, Brazilian National Cancer Institute (INCA), Rio de Janeiro, 20231-050 Brazil; 2 Division of Clinical Research and Technological Development of the National Cancer Institute José Alencar Gomes da Silva (INCA), Rio de Janeiro, RJ Brazil; 3 Immunology and Tumor Biology Program-Research Coordination, Brazilian National Cancer Institute (INCA), Rio de Janeiro, Brazil; 4 Presidency of Research and Biological Collections (VPPCB), Oswaldo Cruz Foundation (FIOCRUZ), Rio de Janeiro, Brazil; 5 Molecular Carcinogenesis Program, Brazilian National Cancer Institute (INCA), Rio de Janeiro, Brazil; 6 Department of Oral Pathology and Oral Diagnosis, School of Dentistry, Universidade Federal do Rio de Janeiro, Rio de Janeiro, Brazil; 7 Experimental Medicine Research Cluster (EMRC), University of Campinas (UNICAMP), Campinas, 13083-970 Brazil; 8 Latin American Cooperative Oncology Group (LACOG)-Head and Neck, University College London (UCL), London, UK

**Keywords:** Oral cancer, Oral pathology, Immunological disorders

## Abstract

To investigate similarities in the gene profile of Oral Lichen Planus and Oral Squamous Cell Carcinoma that may justify a carcinogenic potential, we analyzed the gene expression signatures of Oral Lichen Planus and Oral Squamous Cell Carcinoma in early and advanced stages. Based on gene expression data from public databases, we used a bioinformatics approach to compare expression profiles, estimate immune infiltrate composition, identify differentially and co-expressed genes, and propose putative therapeutic targets and associated drugs. Our results revealed gene expression patterns related to processes of keratinization, keratinocyte differentiation, cell proliferation and immune response in common between Oral Lichen Planus and early and advanced Oral Squamous Cell Carcinoma, with the cornified envelope formation and antigen processing cross-presentation pathways in common between Oral Lichen Planus and early Oral Squamous Cell Carcinoma. Together, these results reveal that key tumor suppressors and oncogenes such as *PI3*, *SPRR1B* and *KRT17*, as well as genes associated with different immune processes such as *CXCL13*, *HIF1A* and *IL1B* are dysregulated in OLP.

## Introduction

Oral Lichen Planus (OLP) is a chronic inflammatory disease clinically characterized by six distinct subtypes that can be seen individually or in combination: white reticular striations, papular, plaque-like, erythematous erosions, ulcerative, and bullous forms. Of all the presentations, the reticular form is the most common, exhibiting a delicate white banding, called Wickham's striae^1,2^. Histologically, OLP is characterized by vacuolar degeneration, a band-like dense inflammatory infiltrate of T lymphocytes at the epithelial-stromal junction, and hyperkeratosis or parakeratosis^3^.

The World Health Organization (WHO) defined in 2017 that Oral Lichen Planus (OLP) is an *oral potentially malignant disorder*, with a possible progression to Oral Squamous Cell Carcinoma (OSCC)^2,4^⁠. Oral lichen planus is estimated to affect up to 2% of the general population. Studies on the potential for malignant transformation observed a transformation rate of 1.14%^5^. Different studies have searched for genetic markers or similarities in the expression profile of genes that participate in the malignant transformation of OLP into OSCC^6–9^. However, the potential for malignant transformation remains the subject of much analysis and remains unclear.

In this study, we demonstrate a repertoire of pathways that have a similar expression profile between OLP and OSCC and that participate in inflammatory processes associated with cancerization. We also explore potential new therapeutic targets and propose drugs that can interact with them and reverse expression changes.

## Results

### Gene expression profiles among OLP and OSCCs

By evaluating batch-corrected samples using PCA analysis, it was observed that OLP samples are more similar to normal oral mucosa samples, however, it is important to note that one of the samples showed high similarity with OSCC (Supplementary Fig. S1). Differential expression analysis revealed a total of 107 DEGs for OLP (83 overexpressed, 24 underexpressed), 331 for early stage OSCC (esOSCC; 182 overexpressed, 149 underexpressed), and 282 for advanced stage OSCC (asOSCC; 96 overexpressed, 186 underexpressed) when comparing to normal samples (Tables S1–S3; Supplementary Fig. S2A–C). Enriched pathways in OLP were related to keratinization, extracellular matrix (ECM) and non-integrin membrane-ECM interactions, as well as immunity-related pathways such as complement, interleukin 10 (IL-10) signaling, antimicrobial peptides, antigen presentation, among others (Supplementary Table S5). In es- and asOSCC, enriched pathways included keratinization, multiple interleukin and interferon signaling pathways, among others (Tables S6, S7).

**Figure 1:**
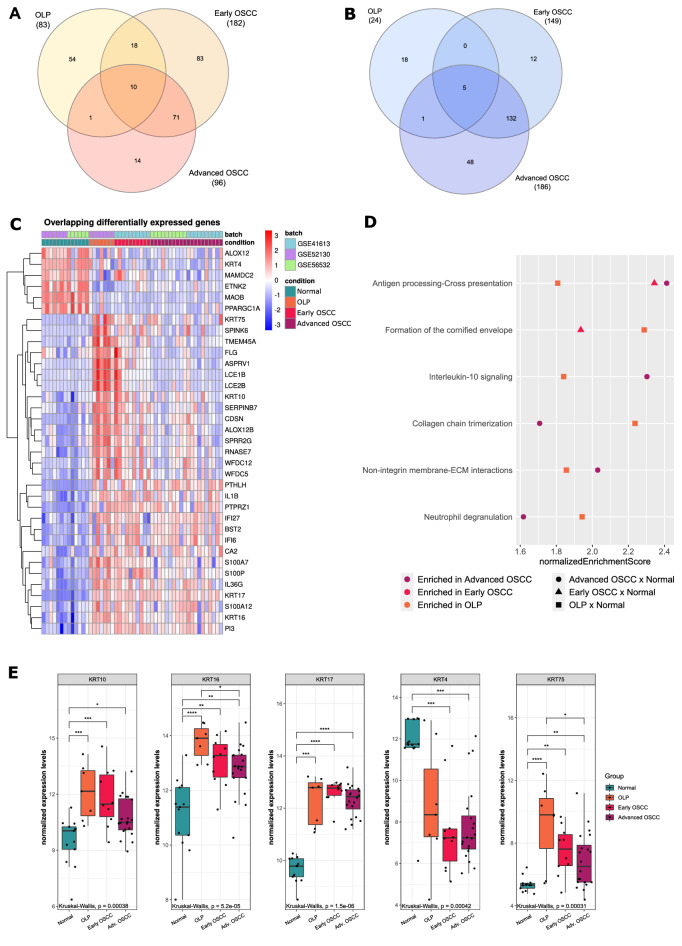
Differentially Expressed Genes between OLP, early, and advanced OSCC microarray datasets compared to normal oral tissue. (**A**) Venn diagram of overlapping DEGs of overexpressed genes. (**B**) Venn diagram of overlapping DEGs of underexpressed genes. (**C**) Clustered heatmap of DEGs shared between OLP, early, and advanced OSCC, as well as overexpressed genes shared by OLP and early OSCC. (**D**) Gene set enrichment analysis (GSEA) of Reactome pathways in Oral Lichen Planus (OLP), early, and advanced Oral Squamous Cell Carcinoma (OSCC). All pathways are up-regulated in comparison with normal tissue. For each pathway, orange squares, red triangles, and purple circles correspond to the normalized Enrichment Score for said pathway in OLP, early OSCC, and advanced OSCC, respectively. Only pathways enriched simultaneously in OLP and at least one of the OSCC stages are represented. (**E**) Differentially expressed keratin genes between OLP and both OSCC groups compared to normal tissue. Box plots represent the normalized expression distribution in each group. Comparisons among groups were made using the Kruskal–Wallis test followed by Dunn's post-hoc test, with p-values lower than 0.05 considered significant for both tests. *p < 0.05; **p < 0.01; ***p < 0.001; ****p < 0.0001.

Among the 35 overlapping DEGs between OLP and OSCC (29 up-regulated and 6 down-regulated), 15 were consistently dysregulated across all comparisons (10 overexpressed, 5 underexpressed) (Fig. 1A,B; Supplementary Table S4). Most of the overlapped genes (94.3–33/35) occurred between OLP and esOSCC, 18 of which were shared only between these two conditions (51.4% of the total of shared DEGs).When using a non-supervised clustering approach based on the expression of the 35 genes signature, all OLP samples were clustered with OSCC samples, mainly in the early stage (Fig. 1C). Of note, genes related to keratinocyte differentiation such as *S100A7*, *S100P*, and *S100A12* as well as immunity-related such as *IL1B*, *IL36G*, *IFI6*, and *IFI27* were also identified, all of which were overexpressed in OLP samples (Fig. 1C).

The enrichment analysis revealed that OLP has six main pathways in common with es- or asOSCC: antigen processing cross-presentation; formation of the cornified envelope; interleukin-10 signaling; collagen chain trimerization; non-integrin membrane-ECM interactions; and neutrophil degranulation, with the antigen presentation pathway enriched in all conditions. Interestingly, antigen presentation and formation of the cornified envelope were the only common pathways between esOSCC and OLP, with the latter being common only between these two groups (Fig. 1D). Some of the DEGs shared by OLP and OSCC are those coding for keratins, including the down-regulation of *KRT4* and up-regulation of *KRT16*, *KRT17*, *KRT10*,and *KRT75* in comparison to normal samples (Fig. 1E). Similar results were found during our validation analysis for *KRT4* and *KRT75* expression using microarray data (Supplementary Fig. S3A,B) RNA-Seq data obtained from TCGA for esOSCC and asOSCC also showed lower *KRT4* expression and higher *KRT17* expression, also consistent with our analysis (Supplementary Fig. S3C).

### Immune microenvironment composition of OLP and OSCC

**Figure 2:**
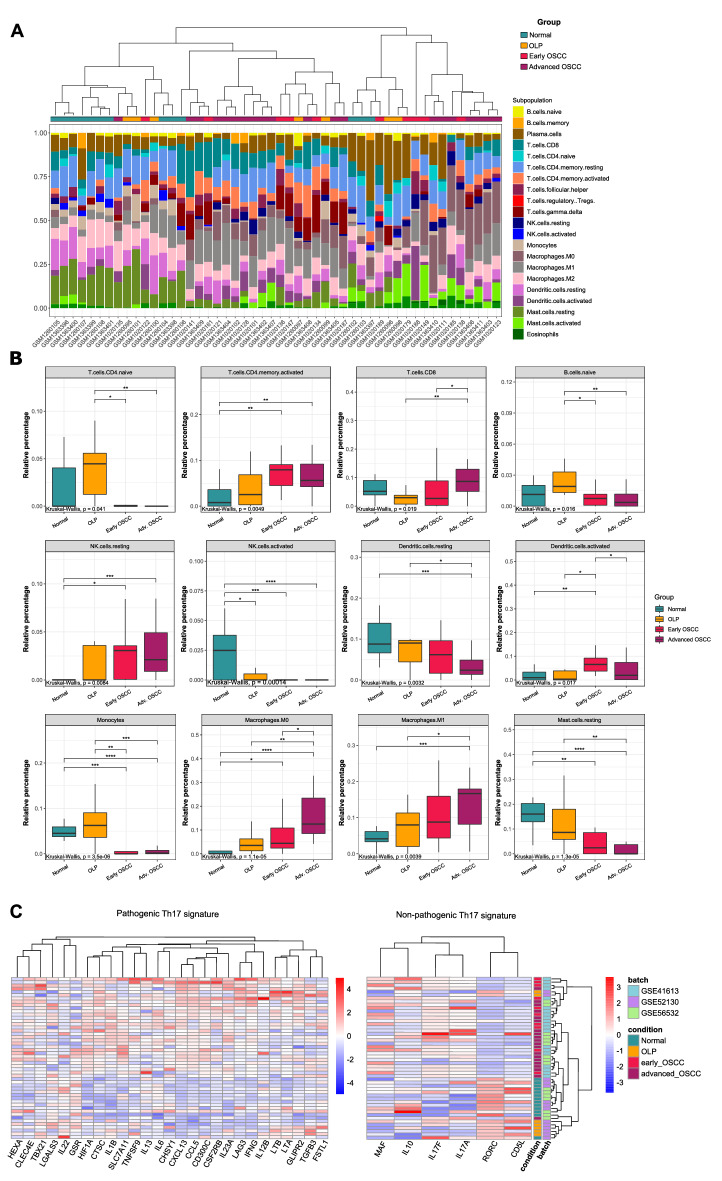
Proportion of cell population signatures identified using CIBERSORTx. (**A**) Stacked barplot showing cell population proportions in each sample (Oral normal mucosa, OLP, early OSCC, advanced OSCC). Dendrogram represents Ward clustering of the samples. (**B**) Relative percentage of each immune cell from oral normal mucosa, OLP, early and advanced OSCC. Comparisons among groups were made using the Kruskal–Wallis test followed by Dunn's post-hoc test, with p-values lower than 0.05 considered significant for both tests. *p < 0.05; **p < 0.01; ***p < 0.001; ****p < 0.0001. (**C**) Clustered heatmap of Th17-related genes. Genes were separated based on non-pathogenic and pathogenic Th17 phenotype-related expression profiles.

Given that OLP is a disease with significant participation of the immune system and both immune evasion and tumor-promoting inflammation are hallmarks of cancer^10^, we have sought to identify the most prominent proportions of infiltrating cells in both conditions. We have used a method that estimates the abundances of cell populations by deconvolution of gene expression data. When clustering samples based on cell population composition, OLP mostly clustered with normal samples, although some of these showed high similarity with the immune cells composition found in OSCC, which are enriched in plasma cells, memory activated CD4+ T cells, resting NK cells and M0 macrophages (Fig. 2A).

The proportions of activated NK cells in OLP, es- or asOSCC samples were significantly lower than the proportions found in the normal oral mucosa. The proportions of CD8+ T lymphocytes, M0 and M1 macrophages in asOSCC showed significantly higher values when compared to OLP. The opposite was observed in resting Mast cells, naïve B cells and monocytes with reduced proportions in both OSCC when compared to OLP and normal oral mucosa (Fig. 2B).

Considering the immune infiltrate populations in both validation datasets, significantly reduced values of activated NK cells were also observed in OLP. Similarly, monocyte and resting mast cell proportions were consistent with our analysis for OSCC, while M0 macrophages showed elevated proportions in asOSCC in the microarray dataset and es- and asOSCC in the RNA-Seq data, consistently to the proportions in the discovery dataset (Supplementary Fig. S4).

Genes related to Th17 cell differentiation, such as *IL1B, CCL5* and *CXCL13*, were differentially expressed in OLP and both stages of OSCC (Tables S1–S3). Therefore, we decided to investigate the genes involved in its differentiation, given the role of Th17 cells in maintaining mucosal immunity homeostasis. OSCC showed high expression of genes related to the pathogenic Th17 signature, while OLP and normal mucosa had high levels of non-pathogenic Th17 cell profile expression. Of note, some genes related to pathogenic signaling were differentially modulated in OLP, such as *CTSC, HIF1A, IL1B, LTA, LTB* and *TGFB3* (Fig. 2C). Additionally, *CTSC*, *HIF1A*, and *IL1B* also show higher expression levels in both stages of OSCC, with an increasing pattern (Supplementary Fig. S5A). The validation data also revealed significant high levels of expression of the *CTSC, LTA*, and *TGFB3* genes in OLP (Supplementary Fig. S5B). Regarding the OSCC groups, however, only *CTSC* and *LTB* exhibited similar expression patterns in comparison to the discovery dataset (Fig. S5C,D). *LTA* showed different patterns, with lower expression in the microarray dataset (Supplementary Fig. S5C) and higher expression in the TCGA validation dataset (Supplementary Fig. S5D).

### Co-expression and network analysis

**Figure 3:**
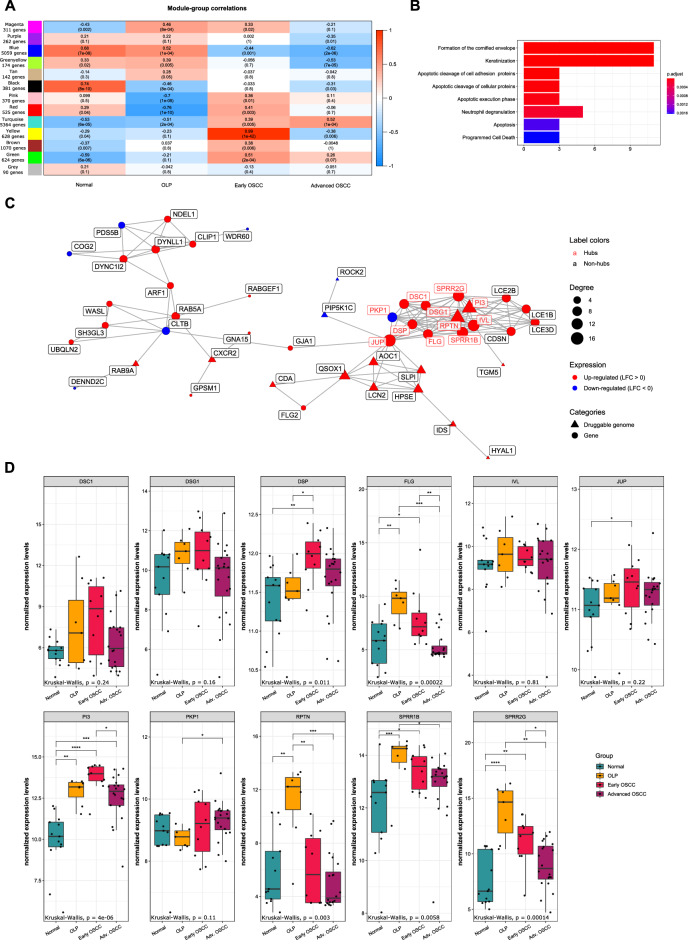
Analysis of co-expressed genes and their regulation. (**A**) Correlations between the co-expression modules and each of the sample groups (normal tissue, OLP, early OSCC, and advanced OSCC). Cell colors correspond to Pearson correlation values between each co-expression module (rows) with groups (columns), from blue (100% inverse correlation) to red (100% direct correlation). Numbers in each cell refer to Pearson's correlation r values, with p-values represented below in parentheses. Correlations with p < 0.05 were considered statistically significant. (**B**) Pathway enrichment of genes in the magenta module using ORA. All represented pathways are significantly enriched (FDR > 0.05). Bar length and colors indicate gene counts and BH-adjusted p-values, respectively. (**C**) Representations of the magenta module as a PPI network (largest connected component). Node size, shape, and color represent degree, category, and LFC in OLP, respectively. Red node labels indicate hubs. (**D**) Hub genes from the magenta module compared between OLP, both OSCC groups, and normal tissue. Box plots represent the normalized expression distribution in each group. Comparisons among groups were made using the Kruskal–Wallis test followed by Dunn's post-hoc test, with p-values lower than 0.05 considered significant for both tests. *p < 0.05; **p < 0.01; ***p < 0.001; ****p < 0.0001.

In addition to differential expression analysis, we have also constructed co-expression gene modules using WGCNA to investigate the connection strength between genes with similar expression patterns and identify potentially co-regulated genes associated with the potential malignization process in OLP. By analyzing a total of 15,000 genes, 12 co-expression modules were obtained, each identified by a color: magenta (311 genes); purple (262 genes); blue, (5059 genes); greenyellow (174 genes); tan (142 genes); black (381 genes); pink (370 genes); red (525 genes); turquoise (5364 genes); yellow (628 genes); brown, (1070 genes); green (624 genes); and grey (90 genes with no co-expression patterns) (Fig. 3A).

To better understand the relationship between OLP and OSCC, we have investigated which co-expression modules had a similar correlation to both conditions simultaneously. For each module, the Pearson correlation coefficient of the module eigengene to the sample groups was calculated. None of the identified modules had a simultaneous significant correlation with OLP and OSCC. Interestingly, only the magenta module showed a significant and positive correlation with OLP (Pearson's correlation r = 0.46; p < 0.0001) and esOSCC (r = 0.33; p = 0.02). Co-expressed genes belonging to this module were mainly associated with keratinization and the formation of the cornified envelope (Fig. 3B).

Co-expression modules, while grouping correlated genes, offer only a glimpse of their dynamic in the cells. To understand how genes in the magenta module interacted, we searched for PPI data in the STRING database and built a network (Fig. 3C).

We identified 11 hubs, 15 transcription factors, 6 clinically actionable genes and 112 members of gene families in the druggable genome, with some of those genes classified in more than one category (Supplementary Table S8). Additionally, drug-gene interactions were identified using all genes in the module. A total of 89 drugs were identified, which interacted with 3 of the 11 hubs (Supplementary Table S9).

The hubs’ expression levels were compared among conditions, with *PI3* being the only gene significantly up-regulated in OLP and all OSCC stages compared to the normal mucosa. Additionally, *FLG*, *SPRR1B*, and *SPRR2G* were significantly up-regulated in OLP and esOSCC, while *DSP* and *JUP* showed higher expression levels only in esOSCC and *RPTN* was up-regulated only in OLP. The four remaining hubs (*DSC1*, *DSG1*, *IVL*, and *PKP1*) didn’t exhibit significant differences in expression compared to the normal mucosa (Fig. 3D).

The hubs with known drug-gene interactions are *PI3* (up-regulated in OLP and OSCC), *IVL* (up-regulated in OLP and esOSCC), and *DSP* (up-regulated in OLP and OSCC) (Fig. 3C,D, Supplementary Table S9).

### Expression drug-response analysis and drug repositioning evaluation

Of 89 drugs identified based on the network’s hub genes, 70 were tested in cancer and 67 were approved in their respective clinical trials. Of these, only 2 were already tested in OSCC (Cisplatin and Sunitinib) and none was approved for use in this type of cancer (Supplementary Table S9). Considering the drugs identified based on the DEGs using the L1000 CDS2 tool, 6 of the 42 drugs were tested and approved for use in cancer. None of them were tested in OSCC. Furthermore, none of the drugs identified in both analyses have been tested in OLP (Supplementary Table S10).

**Figure 4:**
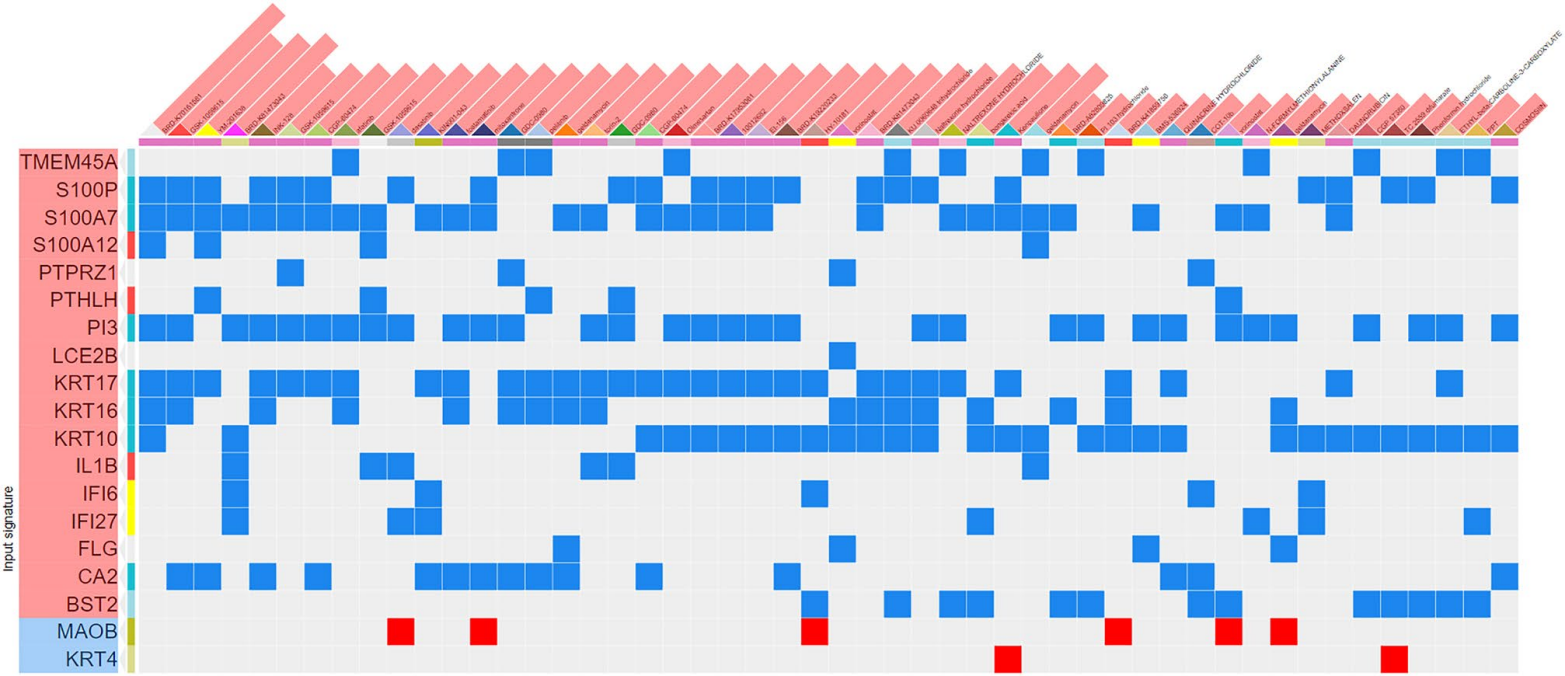
Potential drug target search from shared DEGs using the L1000 CDS^2^ tool on the LINCS Program platform. Genes are represented in rows and drugs in columns, with overexpressed genes colored red and underexpressed genes in blue. Colored squares represent drug-gene interactions, with blue cells representing inhibition and red cells representing gene activation.

Additionally, we searched for drugs that could revert the gene expression phenotype seen in OLP samples. The top fifty matched signatures, corresponding to 42 drugs, were identified based on perturbation data for nineteen of the overlapping DEGs. Six (14.3%) of these drugs are already in use in the clinic or tested in clinical trials, as indicated in Supplementary Table S10. Among the predicted drugs the most represented class was that of the PI3K/mTOR pathway inhibitors, which includes INK-128, GSK 1059615, GDC-0980, Torin-2, KU 0060648 Trihydrochloride, AZD-8055, and PI103 Hydrochloride. The signature of some genes has been reversed by a large number of drugs in the LINCS L1000 cells' signatures such as *PI3* (32/42 drugs), *KRT17* (30/42), *KRT10* (29/42), *S100A7* (29/42), and *S100P* (23/42) (Fig. 4).

## Discussion

Few studies have compared OLP with OSCC using high-throughput data^11,12^ and, to the best of our knowledge, no studies have simultaneously analyzed OLP and OSCC progression and the relationship between this and the inflammatory process of cancerization.

The search for biomarkers that signal the potential for cancerization in OLP has been the subject of many studies. Different genes and proteins have been identified through this process, which include apoptosis modulating proteins (such as survivins), cell cycle regulators (PCNA; P21; P53), tissue remodeling factors (Cathepsin B and MMPs), adhesion molecules (CD44; CK-19, CD34) among others^13–19^. However, most studies do not consider investigating the comparison between OLP and OSCC in different stages, indicating that some important genes or proteins related to this transition may be neglected.

The differential expression of keratin-related genes and the differentiation of keratinocytes in OLP and OSCC may be related to the hyperkeratosis commonly seen in both pathologies. Additionally, genes related to the cornified envelope formation pathway, in addition to being up-regulated in OLP and esOSCC, are co-regulated with genes related to keratinization, corroborating what was demonstrated by Shimada and colleagues^20^. Among the genes, *PI3*, which has already been identified as a molecular biomarker of OSCC samples, stands out (significantly up-regulated in the three conditions), along with *KRT17*^21^. *PI3* was also identified as belonging to the druggable genome, which opens possibilities for use as a target to develop new compounds. In addition, it is interesting to note that the *SPRR1B*, *SPRR2G* and *FLG* genes were significantly up-regulated in OLP and esOSCC, but not in asOSCC. These genes have been up-regulated and associated with tumor development in OSCC and OLP cases^22,23^.

Although not significantly differentially expressed in OLP samples from our validating dataset, which may be related to the low number of samples available, the up-regulation of *KRT17* may also play an important role associated with the carcinogenic process. *KRT17* may be associated with tumor progression by stimulating multiple signaling pathways in OSCC^24^ and has been also linked to proliferation, migration, growth, and metastasis of esophageal squamous cell carcinoma cells in vitro and in vivo^25^. Similar results were observed in non-small cell lung cancer^26^. Interestingly, *KRT17* is one of the main therapeutic targets found in our analyses. Based on these results, it is suggested that *KRT17*, and the related pathways, are important for studying the pathology and malignancy potential of OLP.

The contrast observed in the expression levels of *KRT4* and *KRT10* (down-regulation and up-regulation) may be related to changes in epithelial morphology as reported by Sakamoto and colleagues^27^. Therefore, the authors suggest that *KRT4* may serve as a diagnostic biomarker for OSCC. The same was suggested by Schaaij-Visser and colleagues when revealing that the low expression of *KRT4* in samples from patients with head and neck squamous cell carcinomas (HNSCC), including OSCC samples, may serve as a screening biomarker for local recurrence risk and allow selection for adjuvant treatment or tertiary prevention studies^28^. *KRT4* dysregulation could lead to the overexpression of other keratins, such as *KRT17*. Similar results were observed in OLP lesions, which generally affect the non-masticatory mucosa (such as the bilateral buccal mucosa), with a shift in keratin expression observed by an increased expression of *KRT10* and reduced expression of *KRT4*^29^. These data were in agreement with our analyses on the reduction of *KRT4* in OLP. However, further investigation is needed regarding *KRT10*, because although the validation analyses suggest an increase in its expression, the difference in expression is not significant, in disagreement with what was observed in our analysis.

Despite the results obtained regarding the antigen presentation pathway, further investigations are needed, mainly regarding the nature of the antigen that triggers the pathogenesis of the OLP, which remains unexplained.

Although previous studies have shown that the immune infiltrate profile in OLP is predominantly composed of CD8+ and CD4+ T cells, our analyses have demonstrated a significantly reduced proportion of CD8+ T lymphocytes and NK cells. Still on the predominant microenvironment components in OLP, we demonstrate an up-regulated signature of the chemokine CXCL-13, which has a dual role in tumorigenesis^30^.

We demonstrated that Th17-related pathogenic pathways were positively correlated with OSCC, which is supported by the findings of Gaur and colleagues^31^. Although we did not obtain similar results for the OLP samples, two of these were grouped with early and advanced OSCC samples suggesting that different OLP samples may have different expression profiles, which may be related to a lower or higher risk of malignant transformation. Also, individual investigations related to the pathogenic Th17 molecular signature in OLP showed high levels for several genes involved in tumor progression such as *TGFB3*, *IL1B*, *HIF1A*, *LTA*,and *LTB*. Wang and colleagues showed that *HIF1A* was up-regulated in OLP and OSCC samples, contributing to changes in the expression of genes involved in adaptation to hypoxia and tumor progression^32^. Additionally, Yang and colleagues demonstrated that the activation of *HIF1A* plays a fundamental role during the malignant transformation of OLP by stimulating the apoptosis of keratinocytes^33^. Besides, as *HIF1A* is also related to increased transcription of *IL1B* by cells of the immune system^34,35^, it is suggested that the increased expression of both may be correlated. Together, these results suggest that OLP may have elements of a tumor-like microenvironment as proposed by Peng and colleagues^4^.

Interestingly, in our drug signature analysis, *IL1B* was suggested as a target for treatment with the drugs such as GSK-1059615, Torin-2, and GDC-0980, which are PI3K/mTOR inhibitors^36^. Only GSK-1059615 has already been investigated in OSCC, being able to reduce the proliferation of OSCC cell lines^37^. In OLP lesions, Ma and colleagues showed this pathway mediates the relationship between T cells and keratinocytes and influences the imbalanced cytokine networks in the immune microenvironment^38^. According to the data presented in this study, four other mTOR pathway inhibitors were identified as candidate drugs (PI-103; INK-128; KU-0060648 and AZD-8055). Among them, PI-103 and INK-128 treatment demonstrated inhibition of cell growth and proliferation of OSCC^39^. Also, AZD-8055 was able to induce autophagy in HNSCC cells^40^. Although corticosteroids are recommended for the treatment of OLP, in this work we demonstrated different pharmacological agents that could assist in the treatment and possibly interfere with the malignancy potential of such lesions.

Although our analyses have uncovered some of the genes and pathways that might suggest a malignization process in OLP, it was difficult to acquire gene expression data for this condition due to the limited availability of public data derived from OLP. Additionally, the scant data don't include crucial clinical details about the samples. It is worth mentioning that the data obtained are from different individuals and do not represent a sample of malignant progression or transformation in the same patients.

Our analyzes demonstrate gene expression under different conditions, not necessarily representing the synthesis of proteins present in them. More investigations are required to better understand how proteins are expressed differently in OLP, esOSCC and asOSCC. However, we were able to highlight important biological similarities between the conditions that could point us towards better understanding the inflammatory process that would lead to cancerization. While discarding the use of the term “lichenoid dysplasia” seems appropriate, when it comes to the inflammatory effect on the basal keratinocytes on OLP, the possibility of dysplastic changes on OLP lesions should be studied further before considering exclusion criteria for OLP^41^.

In conclusion, our analysis revealed that OLP is a pathology that shows proximity to the gene expression profile of OSCC, mainly with esOSCC. We reveal signatures in common with the two conditions that can be important targets for drug treatment, as well as in the development of diagnostic and prognostic strategies for the disease. It is considered that OLP and OSCC have multifactorial etiologies, and the intersections between keratinization and lymphocyte differentiation are interesting potential targets for further investigation.

## Materials and methods

### Datasets

Gene expression data from mRNA microarray experiments were obtained from NCBI’s Gene Expression Omnibus (GEO)^42^ using the GEOquery R package^43^. The datasets used corresponded to accession numbers GSE52130 (Illumina HumanHT-12 V4.0 expression BeadChip array), from which 7 OLP and 7 normal oral tissue samples were used; GSE56532 (Affymetrix Human Gene 1.0 ST Array), consisting of gene expression from 10 advanced OSCC samples and 6 normal oral mucosa samples; and GSE41613 (Affymetrix Human Genome U133 Plus 2.0 Array), from which ten random OSCC samples at stages I and II (samples GSM1020161, GSM1020136, GSM1020149, GSM1020147, GSM1020122, GSM1020134, GSM1020188, GSM1020189, GSM1020138, and GSM1020179), and ten at stages III and IV (GSM1020128, GSM1020141, GSM1020185, GSM1020101, GSM1020121, GSM1020135, GSM1020123, GSM1020111, GSM1020102, and GSM1020187) were selected in order to keep group sizes similar and avoid disproportionately large groups to skew subsequent analyses.

Results were validated using independent microarray expression datasets from GEO as well as RNA-seq expression data from The Cancer Genome Atlas (TCGA). Expression datasets from GEO correspond to accession numbers GSE38616 (7 OLP and 7 normal oral mucosa, measured on the Affymetrix Human Gene 1.0 ST Array platform); and GSE3524 (16 OSCC samples in stages II and IV, two of which were removed due to missing staging information, and 4 normal tissue samples, measured on the Affymetrix Human Genome U133A Array platform). Three outliers from GSE38616 (GSM946266 and GSM946263, OLP, and GSM946254, Normal) and one from GSE3524 (GSM80467, advanced OSCC) were identified using principal component analysis (PCA) and removed. For TCGA samples, raw read counts were downloaded from the Head and Neck Squamous cell Cancer (TCGA-HNSC) project using the TCGAbiolinks R package^44^. Primary tumor and normal samples belonging to the "Other and unspecified parts of tongue", "Base of tongue", "Lip", "Palate", "Gum", "Floor of mouth", "Other and unspecified parts of mouth", and "Oropharynx" sites were used in this step.

### Data integration and cross-platform normalization

Data integration and cross-platform normalization were performed in the discovery datasets according to the methods described in^45^. Files containing probe-level intensity data were downloaded using GEOquery and preprocessed using the appropriate package for each platform (oligo for Affymetrix and beadarray for Illumina). Probe level data was extracted, background corrected, and normalized, with the robust multi-array average (RMA) method used for Affymetrix and neqc for Illumina. Probes were mapped to genes using each platform's annotation, with the resulting matrix containing 16,656 features. Normalized expression for genes with multiple probes was aggregated to mean values. Batch effects were corrected using the ComBat method^46^ implemented in the sva R package^47^, considering dataset of origin and sample type as variables. Samples were grouped using PCA to validate successful batch effect correction.

### Differential expression analysis

Differential expression analysis was conducted using the limma R package^48^. Gene expression from OLP, early OSCC (stages I and II), and advanced OSCC (stages III and IV) were compared to normal samples. Differentially expressed genes (DEGs) were identified based on the following cutoffs: Benjamini-Hochberg (BH)-adjusted p < 0.05 and absolute log2 fold change (LFC) > 2. DEGs from each comparison were overlapped using the InteractiVenn online tool^49^. Heatmaps were constructed using the pheatmap R package^50^, using normalized expression values z-scored across samples. Rows representing genes were clustered using Pearson correlation. Columns representing samples were clustered using the hclust function, with non-supervised hierarchical clustering performed based on sample distance, measured as 1 − r, with r being the Pearson correlation coefficient. For individual genes, boxplots were plotted using the ggplot2 package^51^. Comparisons among groups were made using the Kruskal–Wallis test followed by Dunn's post-hoc test, with p-values lower than 0.05 considered significant for both tests. For RNA-Seq data, expression counts were normalized using variance stabilizing transformation through the DESeq2 R package^52^.

### Pathway enrichment analysis

Gene Set Enrichment Analysis (GSEA) was performed on LFC-ranked genes in each condition using the WebgestaltR package^53,54^. The analysis was performed with 1000 permutations. Pathways with a minimum of 5 genes and false discovery rate (FDR) < 0.05 were selected. Result lists for each group had redundant pathways reduced using Webgestalt’s implementation of the Affinity Propagation algorithm. A subsequent filter kept all gene sets that were enriched in OLP and at least one of the OSCC stage groups. These results were presented in dotplots using the ggplot2 package.

Overrepresentation analysis (ORA) was performed for overlapping and OLP-exclusive DEGs, as well as co-expression modules’ genes using the ReactomePA R package^55^. Pathways with a minimum of 5 genes and FDR < 0.05 were selected, and p-values were corrected using BH^56^. The Reactome database was used for both enrichment analyses^57^.

### Immune infiltration cells analysis

Tumor immune infiltration cells composition was estimated using CIBERSORTx^58^. This tool uses a deconvolution algorithm to estimate immune cell types using gene expression data from samples composed of multiple cells (bulk). Batch-corrected, normalized expression data was used to estimate tumor immune infiltration cells using CIBERSORTx's gene signatures for 22 cell types: naïve B, memory B, plasma cells, 7 T cell types (CD8+ T, naïve CD4+ T, resting CD4+ memory T, activated CD4+ memory T, follicular helper T, Treg, γδ T), macrophages (M0, M1 and M2), resting mast cells, activated mast cells, resting NK cells, activated NK cells, resting dendritic cells (resting DC), activated dendritic cells (activated DC), monocytes, eosinophils, and neutrophils.

Stacked bar plots were generated from relative cell type populations using ggplot2. Samples were clustered using the hclust function. Distances based on 1 − r were used for Ward clustering of samples. Population scores were individually compared between groups using Kruskal–Wallis test, followed by pairwise comparisons using Dunn's test, with significant differences identified by p < 0.05.

Additionally, gene signatures related to pathogenic and non-pathogenic Th17 cells were investigated using a 33-gene signature panel based on a previous characterization of Th17 phenotypes^59^. Gene expression and sample clustering visualizations for Th17 cell signatures were made using the pheatmap R package^50^. Boxplots for the signature’s genes were plotted using the ggplot2 package^51^. Comparisons among groups were made using the Kruskal–Wallis test followed by Dunn's post-hoc test, with p-values lower than 0.05 considered significant.

### Co-expression analysis

Gene co-expression modules were constructed using the Weighted Gene Co-expression Network Analysis (WGCNA) R package^60,61^. The 15,000 genes with the highest median absolute deviation (MAD) were selected from the integrated dataset and used as input. A gene pair similarity matrix was generated based on Pearson correlation and converted to a weighted adjacency matrix by elevating it to a β value of 6. This matrix was used to build a topological overlap (TOM) and a dissimilarity matrix (1 − TOM), which was used to build unsigned co-expression modules with a minimum size of 100 genes. WGCNA's module-trait relationship function was used to calculate correlations between module eigengenes and each of the groups (OLP, early OSCC, advanced OSCC, and normal samples). Correlations were considered significant when | r | ≥ 0.3, and p < 0.05.

### Interaction networks construction and drug-gene interactions identification

Genes in the magenta module were used to build a protein–protein interaction (PPI) network using data from the STRING database, v. 11^62^. Interactions with a confidence score < 0.9 and disconnected vertices were discarded. Hub genes were determined by selecting vertices with a degree over the 9th decile of the network’s degree distribution and comparisons in individual hubs’ expression levels were performed as described in the differential expression section.

Gene categories and FDA-approved, antineoplastic drug-gene interactions for hubs were identified using the DGIdb online tool^63^. Clinically actionable genes, transcription factors, and genes coding for protein families belonging to the druggable genome were identified^64,65^. Information such as LFC, gene categories, degree, and hub status were also added to the network. The graph’s largest connected component was used for visualization. Network manipulation was made using the igraph and tidygraph R packages^66,67^. Network plots were constructed using the ggraph R package^68^.

### Search for expression drug-response for differentially expressed genes

Overlapping DEGs were used to search for drugs able to revert their expression signatures using the L1000 Characteristic Direction Signature Search Engine (L1000CDS2) tool on the Library of Integrated Network-Based Cellular Signatures (LINCS) Program platform^69,70^. Drug-gene combinations were ranked by search score, calculated based on the overlap between input DEGs and signature DEGs, that is, gene sets that follow the same perturbation patterns when interacting with a small molecule, with the top 50 drug signatures presented as output. Additionally, putative drug combinations among the small molecule signatures were estimated using this tool and ranked based on their signature overlaps, with the top 50 combinations provided.

### Drug repositioning opportunities evaluation

Drugs identified both by interactions with hub genes and by investigating expression reversion signatures were evaluated for repositioning opportunities using the repoDB database, which compiles information from clinical trials^71^. Drugs were evaluated for whether they were already tested in OSCC, OLP, or other neoplasms as well as their approval status for clinical use.

## Supplementary Information

Supplementary Figures.

Supplementary Tables.

## Data Availability

All datasets used in this work are publicly available. TCGA Harmonized expression data and clinical data are available at the Genomic Data Commons (GDC) Data Portal https://portal.gdc.cancer.gov/. Microarray datasets are available through NCBI's Gene Expression Omnibus^42^. Dataset GSE52130 is available at the weblink https://www.ncbi.nlm.nih.gov/geo/query/acc.cgi?acc=GSE52130 and was generated in Danielsson et al.^72^. Dataset GSE56532 is available at the weblink https://www.ncbi.nlm.nih.gov/geo/query/acc.cgi?acc=GSE56532. Dataset GSE41613 is available at the weblink https://www.ncbi.nlm.nih.gov/geo/query/acc.cgi?acc=GSE41613 and was generated in Lohavanichbutr et al.^73^. Dataset GSE38616 is available at the weblink https://www.ncbi.nlm.nih.gov/geo/query/acc.cgi?acc=GSE38616 and was generated in Gassling et al.^74^. Dataset GSE3524 is available at the weblink https://www.ncbi.nlm.nih.gov/geo/query/acc.cgi?acc=GSE3524 and was generated in Toruner et al.^75^.
