# Supplementary material for: Oral Lichen Planus and Oral Squamous Cell Carcinoma share key oncogenic signatures
Source: Sci Rep. 2022 Nov 30;12:20645. doi: 10.1038/s41598-022-24801-6 (PMC9712651; doi:10.1038/s41598-022-24801-6)
Supplement: Supplementary file 1 — Supplementary Figures. [file 41598_2022_24801_MOESM1_ESM.pdf]

Supplementary figures

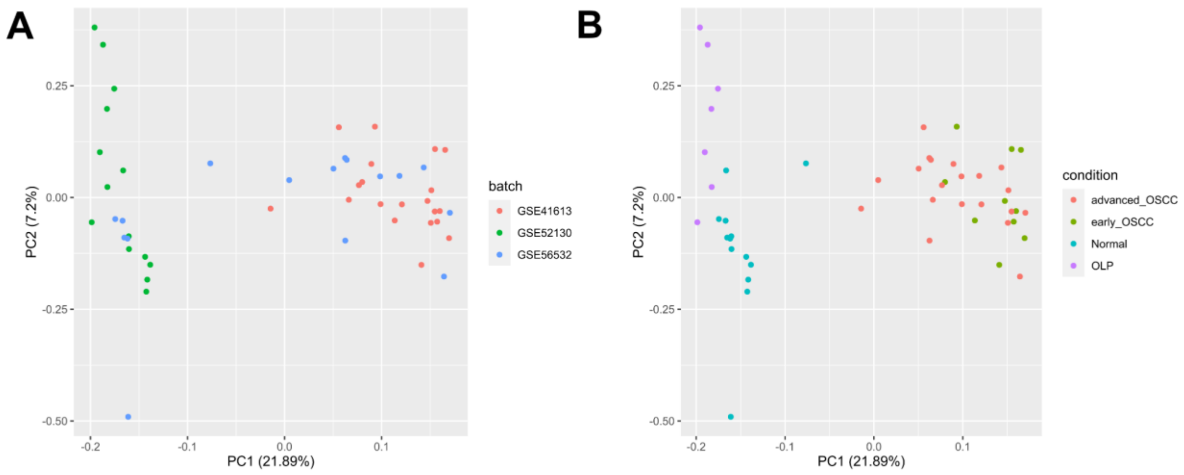

Figure S1. Principal component analysis (PCA) of unified dataset after batch effect correction using ComBat. (A) Samples identified by batch (dataset of origin). (B)

samples identified by group (Normal, OLP, early OSCC, advanced OSCC).

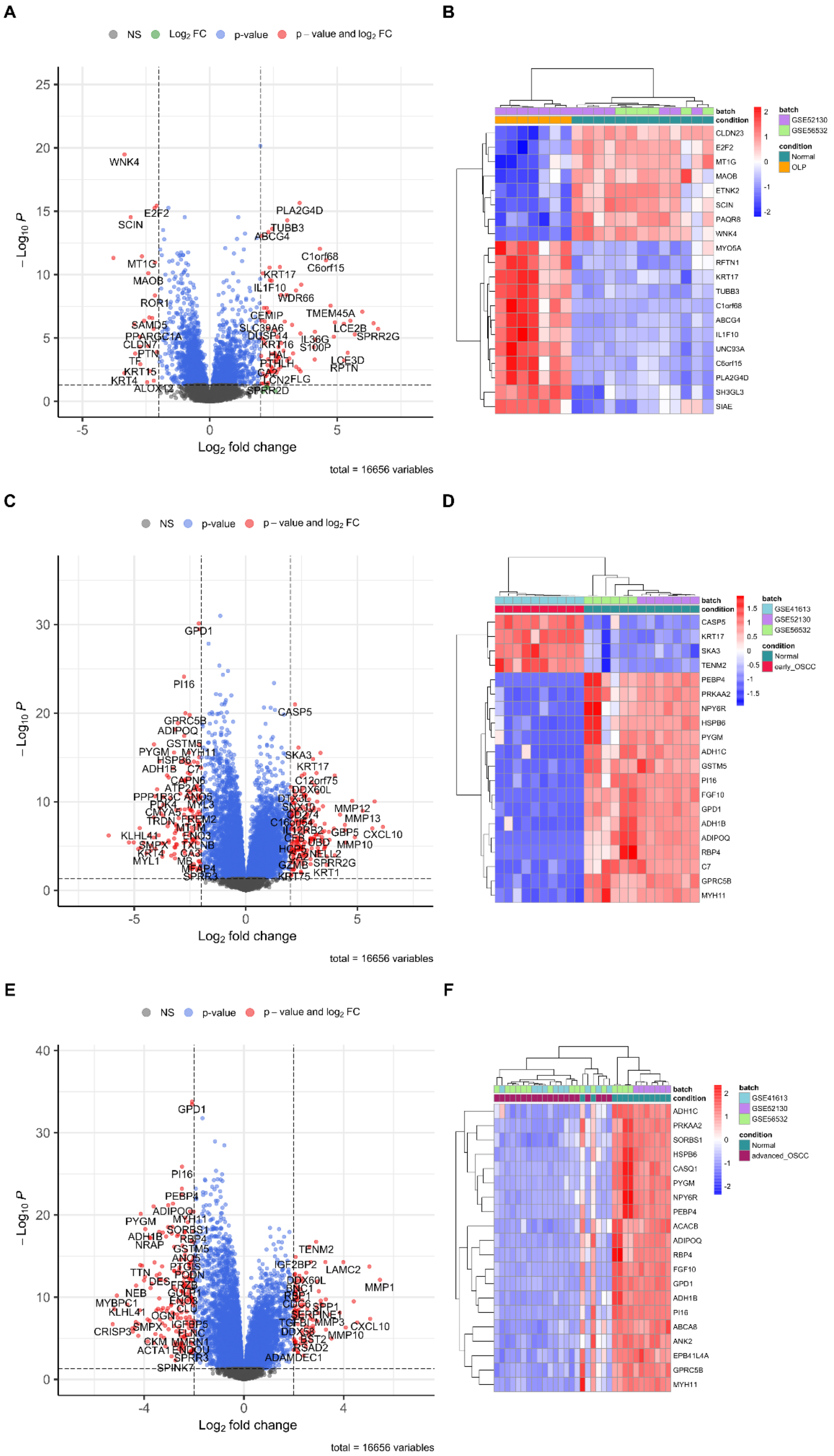
A
NS Log2 FC p-value p – value and log2 FC
– Log10 P
Log2 fold change
total = 16656 variables
WNK4
E2F2
SCIN
MT1G
MAOB
ROR1
SAMD5
PPARGC1A
CLDN7
PTN
TF
KRT15
KRT4
ALOX12
PLA2G4D
TUBB3
ABCG4
C1orf68
C6orf15
KRT17
IL1F10
WDR66
TMEM45A
CEMIP
SLC39A6
DUSP14
LCE2B
SPRR2G
IL36G
S100P
KRT16
HAL
PTHLH
LCE3D
RPTN
CA2
LCN2
FLG
SPRR2D
B
batch
condition
GSE52130
GSE56532
Normal
OLP
CLDN23
E2F2
MT1G
MAOB
ETNK2
SCIN
PAQR8
WNK4
MYO5A
RFTN1
KRT17
TUBB3
C1orf68
ABCG4
IL1F10
UNC93A
C6orf15
PLA2G4D
SH3GL3
SIAE
C
NS p-value p – value and log2 FC
– Log10 P
Log2 fold change
total = 16656 variables
GPD1
PI16
CASP5
GPRC5B
ADIPOQ
GSTM5
PYGM
MYH11
HSPB6
ADH1B
C7
SKA3
KRT17
C12orf75
DDX60L
MMP12
MMP13
CXCL10
MMP10
KLHL41
SMPX
KRT4
MYL1
MB
MFAP4
SPRR3
GBP5
NELL2
GZMB
SPRR2G
KRT1
KRT75
D
batch
condition
GSE41613
GSE52130
GSE56532
Normal
early_OSCC
CASP5
KRT17
SKA3
TENM2
PEBP4
PRKAA2
NPY6R
HSPB6
PYGM
ADH1C
GSTM5
PI16
FGF10
GPD1
ADH1B
ADIPOQ
RBP4
C7
GPRC5B
MYH11
E
NS p-value p – value and log2 FC
– Log10 P
Log2 fold change
total = 16656 variables
GPD1
PI16
PEBP4
ADIPOQ
PYGM
MYH11
ADH1B
NRAP
SORBS1
RBP4
GSTM5
TTN
DES
NEB
MYBPC1
KLHL41
CRISP3
SMPX
ACTA1
CKM
SPINK7
TENM2
IGF2BP2
LAMC2
MMP1
DDX60L
CXCL10
MMP10
ADAMDEC1
SPP1
SERPINE1
RSAD2
F
batch
condition
GSE41613
GSE52130
GSE56532
Normal
advanced_OSCC
ADH1C
PRKAA2
SORBS1
HSPB6
CASQ1
PYGM
NPY6R
PEBP4
ACACB
ADIPOQ
RBP4
FGF10
GPD1
ADH1B
PI16
ABCA8
ANK2
EPB41L4A
GPRC5B
MYH11

Figure S2. Differentially expressed genes in OLP (A, B), early OSCC (C, D), and advanced OSCC (E, F). Volcano plots (A, C, E) represent all DEGs in each group. Dots in red represent genes filtered by both adjusted $p < 0.05$, and $|\log_2 \text{fold change}| > 2$. Clustered heatmaps (B, D, F) represent the top 20 DEGs in each group.

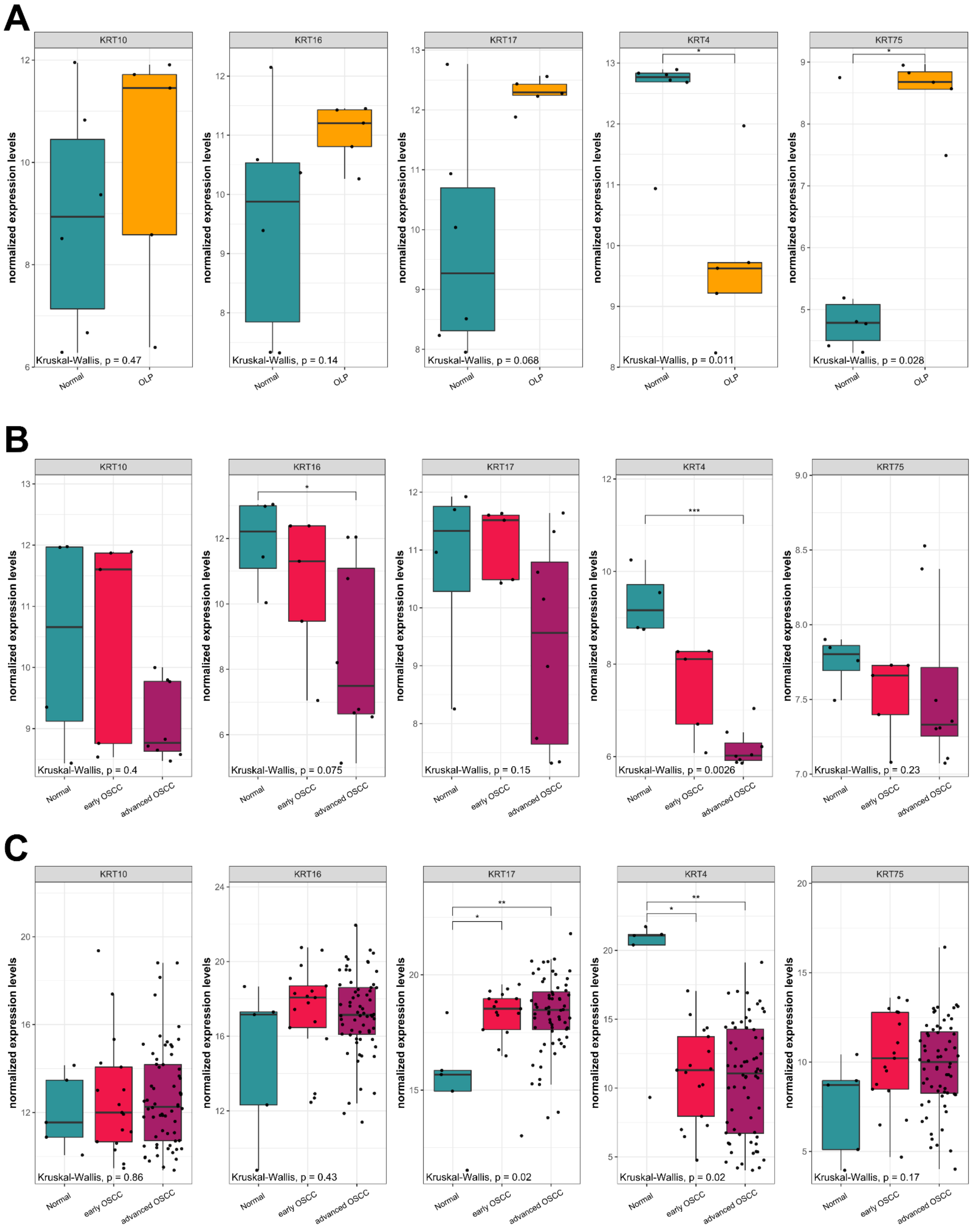

Figure S3. Differentially expressed keratin genes between OLP (microarray dataset GSE38616, (**A)**) and both OSCC groups (microarray dataset GSE3524, (**B)**; and RNA-Seq dataset from TCGA, (**C)**) compared to normal tissue in validation datasets. Box plots represent each group's normalized expression value distributions, with dots representing each sample in the group. Comparisons among groups were made using the Kruskal-Wallis test followed by Dunn's post-hoc test, with p-values lower than 0.05 considered significant for both tests. *, p < 0.05; **, p < 0.01; ***, p < 0.001; ****, p < 0.0001.

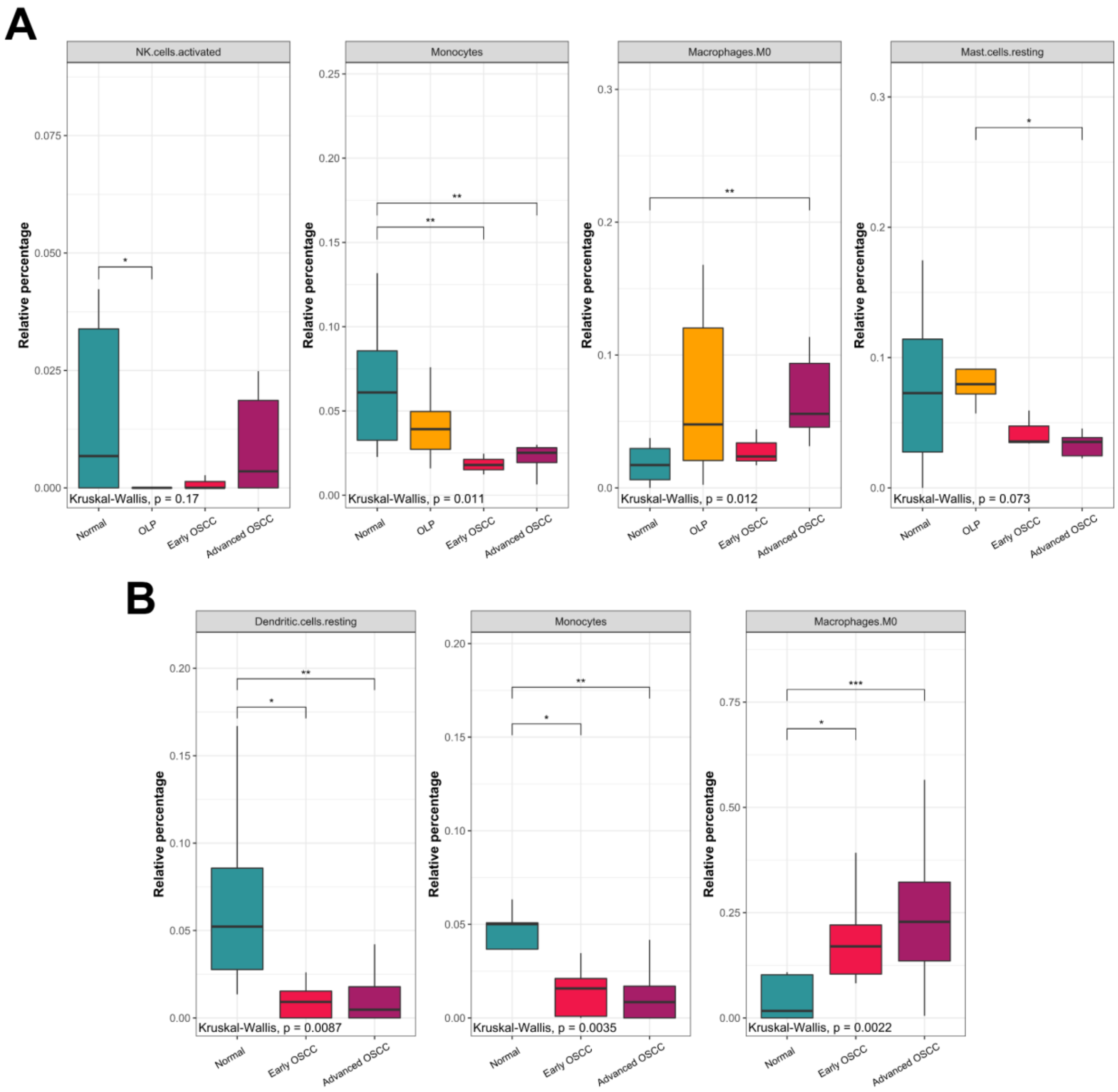

Figure S4. Proportions of cell populations with significant differences in validation datasets. (A) Proportions of activated NK cells, monocytes, macrophages M0, and

resting mast cells, in OLP and both OSCC groups compared to normal tissue (microarray datasets GSE38616 and GSE3524). (B) Proportions of monocytes, macrophages M0, and resting dendritic cells in OSCC samples compared to normal tissue (TCGA dataset). Box plots represent each group's relative proportions of immune infiltrate cells as estimated by CIBERSORTx. Comparisons among groups were made using the Kruskal-Wallis test followed by Dunn's post-hoc test, with p-values lower than 0.05 considered significant for both tests. *, p < 0.05; **, p < 0.01; ***, p < 0.001; ****, p < 0.0001.

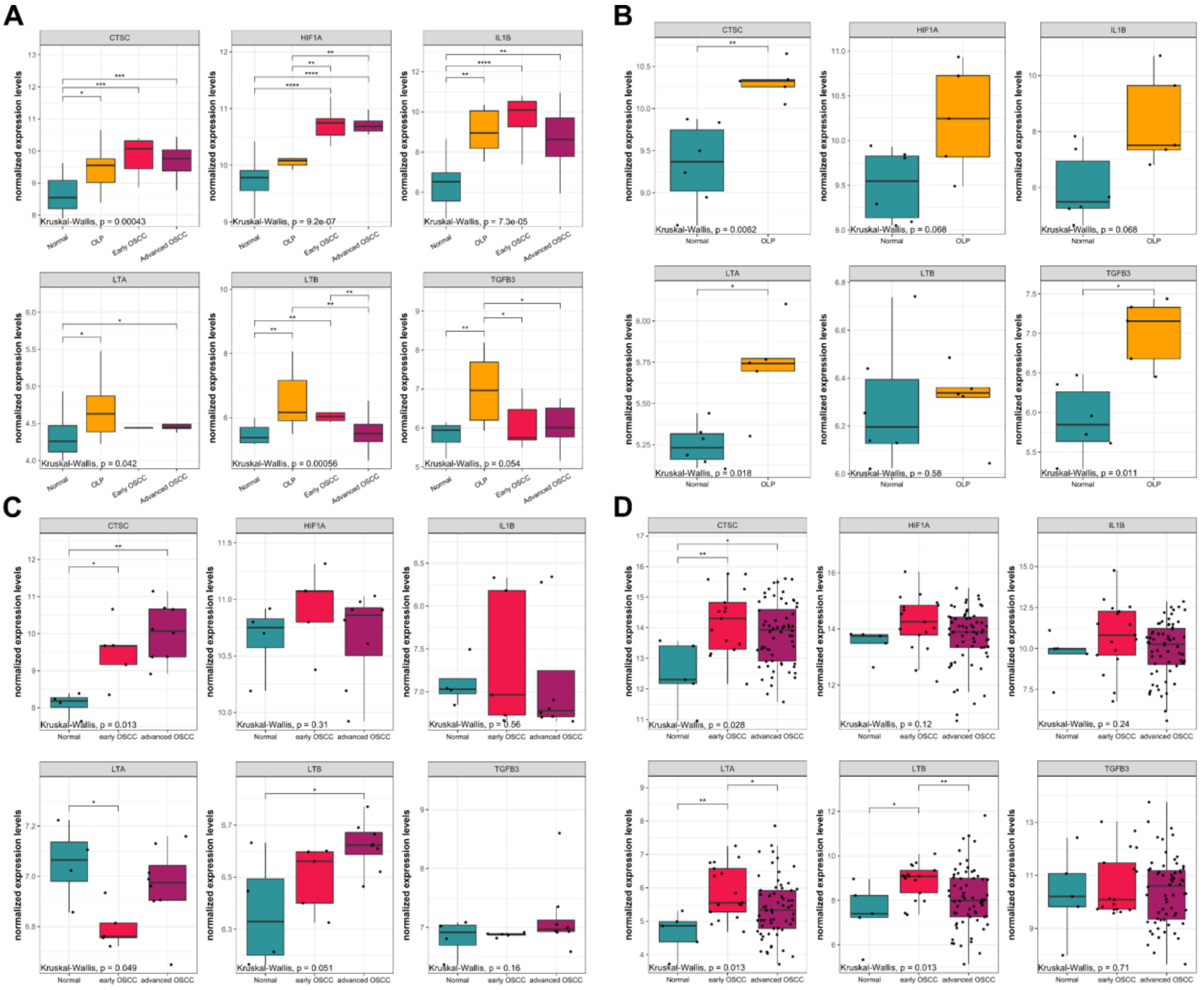

Figure S5. Th17-related genes compared between OLP, both OSCC groups, and normal tissue in discovery (A) and validation datasets (B-D). Box plots represent the normalized expression distribution in each group. Comparisons among groups were made using the Kruskal-Wallis test followed by Dunn's post-hoc test, with p-values lower than 0.05 considered significant for both tests. *, p < 0.05; **, p < 0.01; ***, p < 0.001; ****, p < 0.0001.
